# Differences in Fat Metabolism Between Predicted Survivors And Non-Survivors of Faecal Peritonitis

**DOI:** 10.1186/2197-425X-3-S1-A45

**Published:** 2015-10-01

**Authors:** A Kleyman, W Khaliq, S Neugebauer, M Gräler, M Kiehntopf, M Singer

**Affiliations:** University Hospital Jena, Department of Anesthesiology and Intensive Care Medicine, Jena, Germany; University College London, Bloomsbury Institute of Intensive Care Medicine, London, United Kingdom

## Introduction

A switch from carbohydrate to fat as a fuel source is a hallmark of systemic inflammation. This adaptive reaction allows animals to survive under restricted food supply conditions. However, a prolonged and excessive stimulation of lipid metabolism may be detrimental.

## Objectives

Using our well-characterized 72h fluid-resuscitated rat model of faecal peritonitis (1), where accurate prognostication can be made as early as 6h, we investigated differences in fat metabolism between predicted survivors (S) and non-survivors (NS).

## Methods

Awake, instrumented yet fully mobile male Wistar rats (325 ± 15g) received an i.p. injection of 4µl/g faecal slurry. Fluid resuscitation (50:50 mix of 5% glucose/Hartmann´s; 10ml/kg/h) was commenced at 2h. At 6h, an echo-measured heart rate cut-off of 460 bpm was used to classify animals into predicted S or NS. Control animals were treated identically except for slurry injection. Blood samples were taken for measurement of hormones and metabolites at 6 and 24h. Results were analysed using two-way ANOVA and post-hoc testing, and considered statistically significant when p < 0.05.

## Results

At 6h after sepsis initiation, concentrations of the lipolytic agents, adrenaline and brain natriuretic peptide, were significantly higher in NS (Table 1). The rise in plasma free fatty acids (FFA) was similar in both S and NS. Plasma glucagon and the glucagon:insulin ratio were higher while triglyceride and cholesterol concentrations were significantly lower in NS; this is perhaps explained by glucagon-induced repression of hepatic fatty acid and cholesterol synthesis (2). NS had higher plasma concentrations of ketone bodies and acetylcarnitine, suggesting different utilization of acetylCoA groups between S and NS. The plasma acetoacetate/3-hydroxybutyrate ratio was similar in S and NS at 6h but increased at 24h in S and decreased in NS, reflecting alterations in redox potential and the hepatic NAD^+^:NADH ratio (3).

**Table 1:** Table 1

	Control	Survival	Predicted Non-Survival
Adrenaline	8.56 ± 0.42	9.44 ± 0.23	10.3 ± 0.18
B-natriuretic peptide	118 ± 22	196 ± 35	1318 ± 320a
Glucagon	53 ± 5	62 ± 4	75 ± 5 a
Insulin	0.54 ± 0.25	0.86 ± 0.22	0.87 ± 0.13
Triglycerides	1.22 ± 0.08	1.12 ± 0.04	0.75 ± 0.08 a, b
FFA mg/dL	167 ± 27	514 ± 109	456 ± 122 a
Acetoacetate (AA) µmol/L	91 ± 9	78 ± 57	207 ± 122
3-hydroxybutyrate (3HB)µmol/L	97 ± 8	259 ± 49	355 ± 99 a
Acetylcarnitine µM	9.73 ± 0.40	10.02 ± 0.88	16.75 ± 3.25 a,b

## Conclusions

Predicted non-survivor rats demonstrated a greater stress response to sepsis accompanied by greater alterations in fat metabolism. The consequences of these changes in energy metabolism on the development of organ failure merit further investigation, as this may lead to novel directed therapeutics.

## Grant Acknowledgment

ESICM Basic Science Award, Intensive Care Foundation (UK), NIHR
